# Liver-Metabolizing Genes and Their Relationship to the Performance of Elite Spanish Male Endurance Athletes; a Prospective Transversal Study

**DOI:** 10.1186/s40798-019-0227-7

**Published:** 2019-12-09

**Authors:** David Varillas Delgado, Juan José Tellería Orriols, Carlos Martín Saborido

**Affiliations:** 1 Elite and High-Performance Athletes Research Group, Research Unit, Faculty of Medicine, Universidad Francisco de Vitoria, Pozuelo de Alarcón, Madrid, Spain; 2 Elite and High-Performance Athletes Research Group, Institute of Biology and Molecular Genetics (IBMG/CSIC), University of Valladolid, Valladolid, Spain; 3 Fundación San Juan de Dios. Faculty of Health Sciences San Rafael, Nebrija University, Madrid, Spain

**Keywords:** Physical endurance, Performance, Sports, CYP2D6, Cytochrome p 450, Glutathione transferases

## Abstract

**Background:**

The genetic profile that is needed to define an endurance athlete has been studied during recent years. The main objective of this work is to approach for the first time the study of genetic variants in liver-metabolizing genes and their role in endurance performance by comparing the allelic and genotypic frequencies in elite endurance athletes to the non-athlete population.

**Methods:**

Genotypic and allelic frequencies were determined in 123 elite endurance athletes (75 professional road cyclists and 48 endurance elite runners) and 122 male non-athlete subjects (sedentary). Genotyping of cytochrome P450 family 2 subfamily D member 6 (CYP2D6 rs3892097), glutathione-S transferase mu isoform 1 (GSTM1), glutathione S-transferase pi (GSTP rs1695) and glutathione S-transferase theta (GSTT) genes was performed by polymerase chain reaction (PCR). The combination of the polymorphisms for the “optimal” polygenic profile has been quantified using the genotype score (GS).

**Results:**

Statistical differences were found in the genetic distributions between elite endurance athletes and non-athletes in CYP2D6 (*p* < 0.001) and GSTT (*p* = 0.014) genes. The binary logistic regression model showed a favourable OR (odds ratio) of being an elite endurance runner against a professional road cyclist (OR: 2.403, 95% CI: 1.213–4.760 (*p* = 0.002)) in the polymorphisms studied.

**Conclusions:**

Genotypic distribution of liver-metabolizing genes in elite endurance athletes is different to non-athlete subjects, with a favourable gene profile in elite endurance athletes in terms of detoxification capacity.

## Key Points

- This is the first study that shows that the genetic profile of liver-metabolizing genes in elite endurance athletes (professional cyclists and endurance elite runners) is different from the non-athlete population.
- There is an implication of an “*optimal*” genetic profile in liver-metabolizing genes in systemic recovery from prolonged continuous efforts in this type of endurance sport, favouring sporting performance.
- Elite endurance runners appear to have a more optimal genetic profile in liver-metabolizing genes than professional cyclists.

## 1 Background

The liver performs a variety of unique functions essential for the preservation of homeostasis, including glucose and lipid metabolism, xenobiotic detoxification, and serum protein synthesis. Most of these roles are performed by the hepatocyte, a quiescent and highly differentiated cell expressing a complement of enabling genes [1, 2]. The liver’s central position in systemic metabolism implies a prominent exposure to noxious stimuli derived from environmental toxicants, alcohol, viruses, and dietary habits, the principal causes of liver disease [3].

The combined influence of several genetic variants, each with a significant contribution, as well as the complex interaction of genetic variants, can help to explain individual variations in the human endurance performance. A wide variety of studies find genetic variants that have influence on athletic performance in elite athletes, in running [4–6], soccer [7], triathlon [8], or power efforts [9, 10], finding new candidate genes year by year [11]. Several studies show numerous types of liver-metabolizing genes, referring to their help in the systemic detoxification of drugs and potentially harmful chemicals and cancer inducers [12–16].

The liver is the main organ of cleaning these harmful endogenous products [17], and one of the most striking features that characterize endurance athletes is their faster systemic recovery from continuous efforts, providing improvement in their performance [18, 19]. The probability of a perfect polygenic endurance profile has been previously determined [20], showing the influence of genetic variants in this profile of high sporting performance [8, 21–24]. Recently, the relationship between GSTP gene polymorphisms with performance in Russian and Polish elite athletes has been verified [25], due to a better elimination of exercise-induced reactive oxygen species (ROS).

One of the most striking features that characterize endurance athletes is their faster systemic recovery from continuous efforts, which is mostly related to nutritional supplements like fruit-derived polyphenol [26], quick-absorption carbohydrates [27], and combinations of carbohydrates and proteins [28], providing endogenous improvement performance. In liver metabolism, the interpretation of serum aminotransferases concentration in athletes should consider the release of aspartate aminotransferase (AST) from muscle and of alanine aminotransferase (ALT), mainly from the liver, being markers that predetermine in blood analysis, the endogenous recovery of these endurance athletes [29]. In this work, we approach for the first time the study of genetic variants in liver-metabolizing genes, such as cytochrome P450 family 2 subfamily D member 6 (CYP2D6), glutathione-S transferase mu isoform 1 (GSTM1), glutathione S-transferase pi (GSTP), and glutathione S-transferase theta (GSTT), by comparing the allelic and genotypic frequencies in elite endurance athletes with the non-athlete population.

## 2 Methods

### 2.1 Study Population

The studied population comprised 123 elite endurance athletes (75 professional road cyclists and 48 elite endurance runners) and 122 male non-athlete subjects (sedentary). Non-athlete subjects and elite endurance athletes were of Spanish Caucasian descent. The sample size of the group of endurance elite runners was limited, because in Spain, there is not a high enough number of these athletes who have an elite status compared with the number of professional cyclists. All the elite runners had validated high level and elite sports records in endurance competitions: five athletes ran below 2 h 10 min in marathon, 12 athletes below 1 h 03 min in half-marathon, and the remaining 31 athletes in competitions of 10,000 m and 5000 m ran below 30 min and 14 min, respectively. The athletes participated in marathon or half-marathon of World Championships and/or in 10,000 m and 5000 m runs in the European Championships or Cross-Country World and European Championships. Some of the athletes achieved finalist positions in the marathon and the 10,000 m in the European Championships, with gold and silver medals in the Cross-Country European Championship, representing Spain. The professional cyclists had participated in the Union Cycliste Internationale (UCI) World-Tour events, including Grand Tours, classic cycle races, other one-day races or stage races (often in all of them). Many of them reached one of the top five positions in endurance competitions: Tour de France, Giro d’Italia, and Vuelta a España.

Both runners and cyclists were males, due to the small number of high-level female athletes in Spain who met the inclusion criteria. The non-athlete subjects were males matched by age to athletes; they were not smokers, nor did they suffer from chronic or acute illnesses at the time of sampling.

Informed consent of all the participants in the study was obtained. The protocol of the study was approved by the Committee of Institutional Ethics (University of Valladolid) and agreed with the Declaration of Helsinki for Human Research of 1974 (last modified in 2000).

### 2.2 Genotypes

#### 2.2.1 Target Genes

In order to investigate the role of liver-metabolizing gene variants in the systemic recovery and cleaning of toxic products produced by training and competition in endurance elite sports, the following functional polymorphisms were genotyped in target genes:

c.506-1G>A polymorphism former 1846G>A CYP2D6 gene (location: 22q13.1) generates a change in the canonical sequence at the 3’ end of intron 3. This mutation prevents the splicing of the intron 3 exon 4 junction of the mRNA and codes and inactive protein [30], showing a deficiency of several detoxification enzymes that increase the risk for head and neck squamous cell carcinoma in alcohol- and tobacco-exposed individuals [31].

“*Null*”polymorphism of the GSTM1 gene (location: 1p13.3). This null polymorphism causes the reduction of the detoxification capacity of aromatic hydrocarbons [32, 33] and has been related to predisposition to different diseases, such as liver cancer [34], high risk in patients with clear cell renal cell carcinoma (cRCC) [35], and cardiovascular [36] and respiratory diseases [37, 38].

p.Ile105Val polymorphism of the GSTP gene (location: 11q13). The Isoleucine 105 form exhibited lower catalytic activity towards several carcinogenic diol epoxides as compared with the valine 105 form [39]. Individuals with the GST P1 valine allele showed a significantly higher level of DNA adducts [40]. This decrease in GSTP enzyme activity has been shown to increase the risk of several tumours, like brain [41], myeloid leukaemia [42], lymphomas [43], and gastric cancer [44].

GSTT gene (location: 22q11.23) also has a functional (GSTT*1) and a non-functional allele (GSTT*0). The GSTT can detoxify smaller reactive hydrocarbons, such as ethylene oxide and diepoxy butane. The null genotype of GSTT was reported to be associated with an increased risk of bladder cancer, lung cancer, and myelodysplastic syndrome [45].

#### 2.2.2 Deoxyribonucleic Acid Extraction and Genotyping

##### Nucleic Acid Purification

Genomic DNA was obtained from ethylenediaminetetraacetic acid (EDTA) anti-coagulated blood samples according to standard phenol-chloroform procedures, followed by precipitation with ethanol.

##### Genotyping

GSTM1 and GSTT genotyping were carried out by direct PCR amplification and subsequent agarose gel electrophoresis, as previously described [32, 33, 46, 47]. CYP2D6 and GSTP polymorphisms were genotyped by polymerase chain reaction (PCR) amplification, followed by specific restriction fragment analysis in 2% agarose gel, as previously described [30, 39]. All PCR reactions were carried out in 20 μl of the total volume, being DNA concentrations between 125 and 250 μgr. The primers sequence at target genes and PCR conditions are shown in Table 1 and Table 2.

**Table 1:** Primers sequence at target genes

CYP2D6	Forward	5′-GCCTTCGCCAACCACTCCG-3′
	Reverse	5′-AAATCCTGCTCTTCCGACGC-3′
GSTM1	A	5′-CGCCATCTTGTGCTACATTGCCCG-3′
	B	5′-ATCTTCTCCTCTTCTGTCTC-3′
	C	5′-TTCTGGATTGTAGCAGATCA-3′
GSTP	Forward	5′-ACCCCAGGGCTCTATGGGAA-3′
	Reverse	5′-TGAGGGCACAAGCCCCT-3′
GSTT	Forward	5′-TTCCTTACTGGTCCTCACATCTC-3′
	Reverse	5′-TCACCGGATCATGGCCAGCA-3′

**Table 2:** PCR conditions

CYP2D6		Initial denaturation	94° 5 min
	× 30 cycles	Denaturation	94° 1 min
		Annealing	60° 1 min
		Extension	72° 2 min
		Final extension	72° 5 min
GSTM1		Initial denaturation	95° 5 min
	× 40 cycles	Denaturation	94° 30 sec
		Annealing	58° 30 sec
		Extension	72° 45 sec
		Final extension	72° 8 min
GSTP		Initial denaturation	94° 5 min
	× 35 cycles	Denaturation	94° 30 sec
		Annealing	55° 30 sec
		Extension	72° 30 sec
		Final extension	72° 5 min
GSTT		Initial denaturation	95° 5 min
	× 30 cycles	Denaturation	95° 1 min
		Annealing	60° 1 min
		Extension	72° 1 min
		Final extension	72° 10 min

### 2.3 “Optimal” Polygenic Profile for Endurance Performance in the Spanish Population (Caucasian)

The probability that an individual bears the “*optimal*” genotype for each of the four polymorphisms was calculated based on the typical frequency of each genotype observed in Spanish people (Caucasian descent for the population of ≥ 3 generations) [45, 48] (Table 3). An “*optimal*” GS of 2 was scored for the polymorphisms of the CYP2D6 and GSTP genes and an “*optimal*” GS was scored 1 for the polymorphisms of the GSTM and GSTT genes. A scale was made with the estimated probability of having a “*perfect*” genetic profile, considering the number of polymorphisms included in the entire profile [24].

**Table 3:** Genotyping frequency in the Spanish population and elite endurance athletes

Symbol	Gene	Polymorphism	Genotypes (2 or 1 = “*optimal*” endurance genotype)	Frequency in Spanish population (%) (*)	Frequency in Spanish elite endurance athletes (%)
*CYP2D6*	Cytochrome P450 family 2 subfamily D member 6	c.506-1G>A	0 = AA–1 = GA–2 = GG	4–27–69	1–14–85
*GSTM1*	Glutathione S-transferase *mu*	Functional(+)/null(−)	0 = −–1 = +	82–18	71–29
*GSTP*	Glutathione S-transferase *pi*	Ile(I)105Val(V)	0 = GG–1 = GA–2 = AA	41–45–14	6–31–63
*GSTT*	Glutathione S-transferase *tetha*	Functional(+)/null(−)	0 = −–1 = +	64–36	28–72

(*) www.ensembl.org

Based on the typical frequencies observed from the “*optimal*” genotypes, a scale was generated, estimating the probability of possessing a “*perfect*” genetic profile, having taken into account the polymorphisms included [24].

### 2.4 Polygenic Potential for the Endurance Performance of the Spanish Population

The combined influence of the four polymorphisms studied was calculated, following the procedure of Williams and Folland [20]. First, each genotype was scored within each polymorphism (Table 3). A genotype score (GS) of 2 or 1 was assigned to the “*optimal*” or preferable endurance genotype, while a GS of 0 was assigned to the less optimal genotype [49]. Secondly, the GSs of all genotypes (GS_CYP2D6_ + GS_GSTM1_ + GS_GSTP_ + GS_GSTT_) were added, and finally the score was transformed to a 0–100 scale to facilitate interpretation, namely the total genotype score (TGS), as follows:

*TGS =* (*100/6*) *×* (*GS*_*CYP2D6*_ *+ GS*_*GSTM1*_ *+ GS*_*GSTP*_ *+ GS*_*GSTT*_)

The maximum score for CYP2D6 and GSTP was 2 and for GSTM1 and GSTT it was 1. Thus 6 is the maximum total sum of all GSs, and therefore the “*optimal*” or preferable genotypic profile. As indicated [20], a TGS of 100 represents a “*perfect*” profile and a TGS of 0 should be the “worst” possible profile for endurance sports when all GSs have a score of 0. Finally, the TGSs’ distribution between elite endurance athletes and non-athletes was assessed.

### 2.5 Polygenic Potential for Endurance Performance in the Spanish Control Population and High-Level Athletes

A polygenic profile was calculated for each endurance elite athlete and non-athlete subject, as described, in order to analyse both the nature of the TGS distribution in a highly selected group of Spanish endurance athletes, and the differences between these and the subgroups of cyclists and runners vs. non-athletes.

### 2.6 Statistical Analysis

The statistical average and kurtosis were calculated using Statistical Package for the Social Sciences (SPSS), v.20.0 for Windows (IBM Corp. Released 2012. IBM SPSS Statistics for Windows, Version 20.0. Armonk, NY: IBM Corp). The probability of having an “*optimal*” endurance genotype for one to four polymorphisms between elite endurance athletes and non-athletics was calculated by using the χ^2^ test with fixed α 0.05. The genotypic frequencies of the polymorphisms in CYP2D6, GSTM1, GSTP, and GSTT genes were compared between elite endurance athletes and non-athletics, using a χ^2^ test with fixed α 0.05.

The ability of TGS to correctly distinguish potential elite endurance athletes from non-athletes (0 = non-athlete, 1 = elite) was assessed using receiver operating characteristic (ROC) curves [50]. With that purpose, the area under the ROC curve (AUC) was calculated with confidence intervals of 95% (95% CI). Finally, a binary logistic regression model was used to study the relationship between TGS and the athletic status.

## 3 Results

**Fig. 1:**
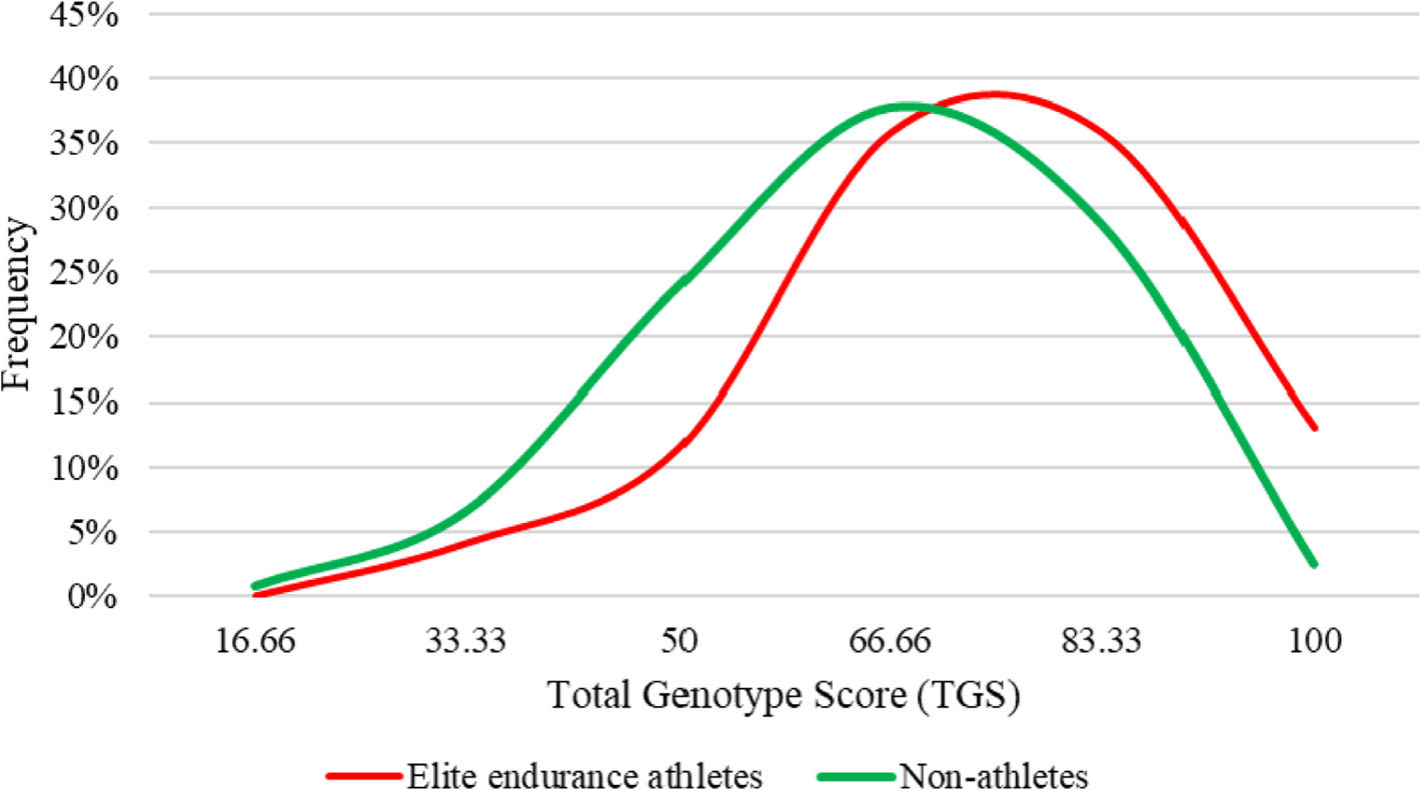
TGS distribution in elite endurance athletes and non-athlete subjects

**Fig. 2:**
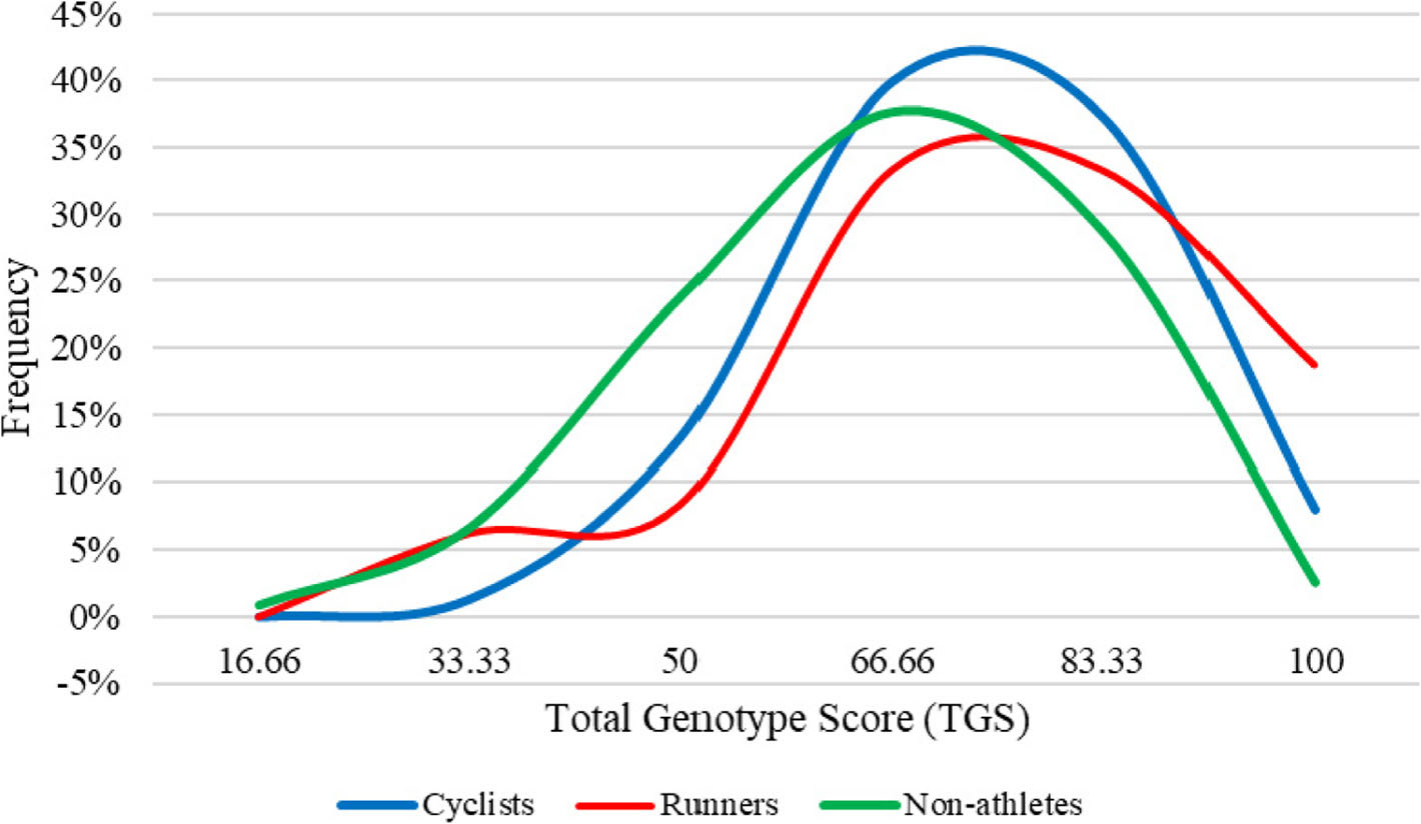
TGS distribution in elite endurance athletes: cyclists, runners and non-athlete subjects

In the non-athlete population, the mean value of the TGS was 65.706 (± 16.360), statistical kurtosis: − 0.182 (± 0.435), and in the group of elite endurance athletes it was 73.709 (± 16.531), statistical kurtosis: − 0.096 (± 0.433). The mean value of the TGS in professional cyclists was 72.885 (± 15.445) statistical kurtosis: − 0.087 (± 0.548), and of endurance elite runners it was 74.996 (± 18.193), statistical kurtosis: − 0.052 (± 0.674). The distributions of TGS frequencies of the 122 non-athletes and 123 elite endurance athletes are represented in Fig. 1. Figure 2 shows the frequency distribution of the TGSs of cyclists and elite runners and the 122 non-athlete subjects.

TGS distribution in elite endurance athletes is shifted to the right with respect to non-athletes. Sixteen elite endurance athletes (13.0%) and only three non-athletes (2.5%) exhibited an “*optimal*” TGS of 100. The difference in the distribution of TGSs between both groups was statistically significant (*p* < 0.001) (Table 4).

**Table 4:** Distribution of elite endurance athletes and non-athletes with GS of 0-6 in target genes

Number of accumulated genotypes in an “*optimal*” GS individual score	Elite endurance athletes (*n* = 123) (accumulative %)	Non-athletes (*n* = 122) (accumulative %)	*p* value
0	0 (0.00%)	0 (0.00%)	< 0.001
1	0 (0.00%)	1 (0.82%)	
2	5 (4.06%)	8 (7.37%)	
3	14 (15.44%)	29 (31.14%)	
4	44 (51.22%)	46 (68.85%)	
5	44 (86.99%)	35 (97.54%)	
6	16 (100.00%)	3 (100.00%)	

**Fig. 3:**
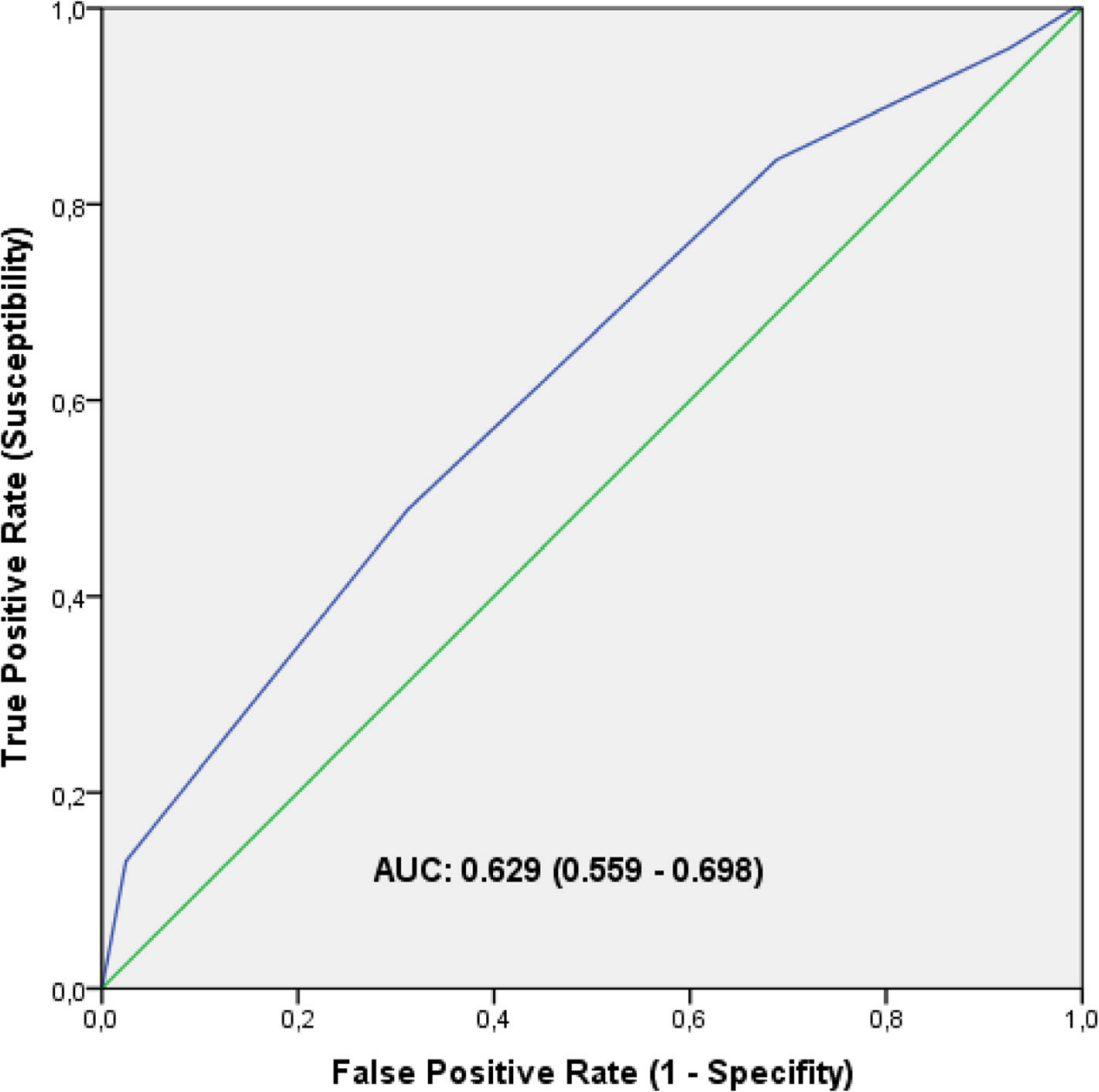
ROC curve summarizing the ability of TGS to distinguish potential elite endurance athletes from non-athletes

ROC analysis showed significant discriminatory accuracy of TGSs in the identification of elite endurance athletes (AUC = 0.629; 95% CI: 0.559–0.698) (*p* < 0.001) (sensitivity = 0.488, specificity = 0.689) (Fig. 3). The corresponding TGS value at this point was 74.995. Binary logistic regression analysis showed that subjects with a higher TGS of this value (74.995) had an odds ratio (OR) of 1.171 (95% CI: 0.816–1.680 (*p* = 0.245)) of being elite endurance athletes, compared to those with a TGS below this value. The endurance elite runners showed an OR at the cut-off point in comparison to the non-athlete population of 2.403 (95% CI: 1.213–4.760) (*p* = 0.002)) and professional cyclists, in comparison to non-athlete subjects, had an OR of 1.029 (95% CI: 0.735–1.442) (*p* = 0.462)).

Genotype distribution of liver-metabolizing genes in the elite endurance athletes’ group when compared with the non-athlete population was statistically significant for CYP2D6 (*p* < 0.001), showing a higher frequency in the “optimal” genotype in athletes (G/G 85.36%) than the non-athlete population (G/G 62.30%); in GSTT “optimal” polymorphism, the frequency was higher in elite endurance athletes than non-athletes’ (*p* = 0.014) (Table 5). Between both groups of elite endurance athletes (cyclists and runners), statistically significant results were found in CYP2D6 (*p* = 0.002) and GSTT genes (p = 0.049) compared with non-athletes (Table 6).

**Table 5:** Genotype distribution in elite endurance athletes and non-athletes of liver-metabolizing polymorphisms

	Elite endurance athletes (*n* = 123) *n* (%)	Non-athletes (*n* = 122) *n* (%)	*p* value
CYP2D6			
A/A	1 (1.66%)	2 (1.64%)	< 0.001
G/A	17 (13.82%)	44 (36.06%)	
G/G	105 (85.36%)	76 (62.30%)	
GSTM1			
+	36 (29.27%)	45 (36.88%)	0.205
−	87 (70.73%)	77 (63.12%)	
GSTP			
G/G	8 (6.51%)	13 (10.65%)	0.122
G/A	38 (30.89%)	48 (39.35%)	
A/A	77 (62.60%)	61 (50%)	
GSTT			
+	89 (72.36%)	70 (57.38%)	0.014
−	34 (27.64%)	52 (42.62%)

**Table 6:** Genotype frequencies in liver metabolizers between elite endurance athletes (cyclist, runners) and non-athletes

		CYP2D6 genotype				GSTT genotype		
		*GG*	*GA*	*AA*	*p* value	*+*	*−*	*p* value
Elite endurance athletes	Cyclists	63 (84.00%)	11 (14.66%)	1 (1.33%)	0.002	54 (72.00%)	21 (28.00%)	0.049
	Runners	42 (87.50%)	6 (12.50%)	0 (0.00%)		35 (72.91%)	13 (27.09%)	
	Non-athletes	76 (62.29%)	44 (36.06%)	2 (1.64%)		70 (57.37%)	52 (42.63%)	

## 4 Discussion

A great variety of external factors influence an individual’s ability to succeed in sport; however, genetics may play an important role in determining sporting achievement, so creating individualized training programmes based on genetic predispositions is important, as is identifying athletes who need an adapted training routine to improve their performance and to account for individual susceptibility to injury [51, 52].

For many years, genes with allelic variants have been identified as predisposing individuals to elite endurance, including Actinin Alpha 3 (ACTN3) [9] and Angiotensin Converting Enzyme (ACE) [53]. A recent study of a cohort of Caucasian elite athletes, from 1500 m runners to marathon runners, showed no differences in endurance running times related to these polymorphisms in ACE and ACTN3 genes previously described [54]. This study presented 698 Caucasian elite athletes with similar performance profile to our sample, found different results from ours. The results should be corroborated in subsequent studies with the same polymorphisms presented in our elite endurance athletes.

Different pathologic as well as non-pathologic conditions could increase the production of free radicals or drain the antioxidant defence system. Prolonged and intensive exercise is one of the oxidative stress-inducing conditions, via overproduction of reactive oxygen species and reactive nitrogen species.

This oxidative stress in endurance sports and elite athletes is a determinant of performance. It is known that in competitions like cycling, in which the accumulated efforts of several weeks affect the performance, which also happens in endurance elite runners, with their requirement of several weeks of preparation for a world championship, European championship or marathon, this is mainly due to the alteration in the redox-system of the systemic homeostasis and withdrawal of toxic products generated by high oxidative stress [55–57]. There is recent evidence that diet has an important role in helping to reduce this oxidative stress by ingesting carbohydrate-rich diets [58, 59] and lipids [60] in long-distance sports, especially cycling [61, 62] and elite running [63, 64]. However, there are still insufficient studies that consider the genetic heritage of individuals and especially high-performance athletes in the systemic cleansing of oxidative stress. Only a recently published pilot study by Al-Khelaifi et al. [65] provides evidence that high-power and high-endurance athletes exhibit a distinct metabolic profile, defined by a genetic pool, that reflects steroid biosynthesis, fatty acid metabolism, oxidative stress, and energy-related metabolites; this will become a broad field of study in the coming years to ascertain the systemic recovery of high performance athletes. Al-Khelaifi et al.’s study analysed 743 metabolites; gamma-glutamyl amino acids were significantly reduced in both high-power and high-endurance athletes compared with moderate counterparts, indicating an active glutathione cycle, the same metabolic pathway that can explain the phenotype of the genotypes showed in this study. To date, the genetic markers and polymorphisms that have been studied on an individual basis have been involved in muscle damage [66], muscular modulation [67–70], and in the immune system of these elite endurance athletes [71, 72]; these have been necessary studies that have shown that all these polymorphisms must be investigated in order to understand the implications of oxidative stress in a global way.

The enzymatic activity of the proteins coded by the sequences of GSTM1 [73] and GSTP [74, 75] genes has been previously identified as a risk factor in diseases of oxidative stress and is associated with the risk of developing chronic severe ethanol liver damage. On the other side, CYP2D6 is a molecule of the cytochrome P450 superfamily that metabolizes several drugs and endogenous molecules. Its activity has been associated with different oxidative stress-related processes, as mitochondrial respiration [75], liver toxicity [76], or toxicity of reactive metabolites in erythrocytes [77]. This work is the first in this field that shows a pool of polymorphisms in liver-metabolizing genes, such as glutathione transferases and the cytochrome P450 family 2 subfamily D member 6 (which influence systemic recovery by the hepatic cleansing of endogenous toxic products generated by intense exercise), between the non-athletic population and elite endurance athletes. A recent study shows the relationship between GSTP polymorphism in Russian and Polish athletes [25], showing statistical data among high-performance athletes and the non-athlete population. But nevertheless, in this study, no differences have been found between athletes and the non-athlete population in the GSTP polymorphism studied, which may be due to sample size (698 athletes in the Zarebska study vs. 122 athletes in this study).

CYP2D6 and GSTT polymorphisms present a genotypic frequency in elite endurance athletes different from the non-athlete population; it is associated with a higher metabolic activity of proteins [30, 45, 46], a fact that predisposes this group to a better metabolic capacity. Differences between the two sub-groups of endurance athletes are not evident, as the frequencies between cyclists and runners are similar, corresponding to a CYP2D6 polymorphism of an “*optimal*” genotype of 84% in cyclists and 87.5% in runners, while null polymorphism in the GSTT gene was “*optimal*” in 72% of cyclists as against 72.91% of runners (Table 6). However, the null genotype of GSTM1 showed more frequently in athletes, needs to be investigated in subsequent studies to verify these Caucasian athletes’ frequencies. In turn, it was found that the definition of “*optimal*” genotypes in the work of Williams and Folland [20] implied that elite endurance athletes have a significantly higher proportion of TGS than the non-athlete population and a lower proportion of not optimal genotypes (*p* < 0.001) (Table 4), showing that the liver-metabolizing genes studied presented in the group of elite endurance athletes an “*optimal*” genotype that was significant in comparison to non-athletes. The endurance elite runners present favourable genetics in these polymorphisms than professional cyclists due to provokes more concentration of oxidative stress biomarkers than cycling [78, 79], using the glutathione (GSH) pathway, corroborated by TGS scores; the endurance elite runners showed an OR at the cut-off point in comparison to the non-athlete population of 2.403 (*p* = 0.002) and professional cyclists with respect to non-athlete subjects showed an OR of 1.029 (*p* = 0.462).

In this research, the genetic profiles defined by genetic polymorphisms of liver-metabolizing genes in 123 elite endurance athletes were compared with 122 non-athlete males. We decided to include these liver-metabolizing genes in the study, since the toxic effects described are similar to those of high-performance sportsmen in continuous efforts, being able to produce the endogenous products as free radicals and peroxides as a decrease in the physical capacity of them. Oxidative stress is the consequence of an impaired balance between free radical production and the endogenous antioxidant protection system. Only four known polymorphisms have been studied, one within each target gene. Another interesting variant within these genes has not been included and constitutes ground for further studies and a better definition about the role of genetic variations in liver-metabolizing genes and endurance performance.

In other previous genetic association studies of sportive performance, the ethnic and geographical origins of the athletes included in the studies have been mixed. Our work does not present these limitations, since we have focused on Caucasian Spanish elite endurance athletes’ performance, provided by the Spanish Higher Council of Sports (CSD).

For the first time, to the best of our knowledge, the relationship between these polymorphisms in liver-metabolizing target genes is shown, leading the capacity of systemic recovery in elite endurance athletes; this is a new type of genetic study, showing a definitive model of the profile in these types of genes that help the capacity of systemic cleansing of ROS produced by the physical effort in this group of subjects in order to understand the multiple and complex mechanisms that define it.

Subsequent studies in relation to genetic profiles and the serum analysis of catabolites for oxidative stress products in elite endurance athletes to determine their ability to clean these products for a return to systemic homeostasis should be carried out in order to corroborate the results shown in this study and to be able to conclude that these genetic markers are predisposed to the metabolizing capacity of toxic waste products induced by high performance endurance.

## 5 Conclusions

It is demonstrated for the first time that genotypic distribution in elite endurance athletes as regards endurance (professional cyclist and elite runners) is different to the non-athlete Caucasian population, there being a favourable gene profile in terms of the detoxification capacity. These results open a new way of study of this genes group to complete the knowledge of oxidative stress and recovery of systemic homeostasis in high performance in endurance sports.

## Data Availability

All data generated or analysed during this study are included in this published article.

## Abbreviations

95%CI: Confidence intervals of 95%
ACE: Angiotensin I-converting enzyme
ACTN3: Actinin alpha 3
ADRB: Adrenergic receptor beta
ALT: Alanine aminotransferase
AST: Aspartate aminotransferase
AUC: Area under the ROC curve
cRCC: Cell renal cell carcinoma
CSD: Spanish Higher Council of Sports
CYP2D6: Cytochrome P450 family 2 subfamily D member 6
DNA: Deoxyribonucleic acid
EDTA: Ethylenediaminetetraacetic acid
GS: Genotype score
GSH: Glutathione
GSTM1: Glutathione-S transferase mu isoform 1
GSTP: Glutathione S-transferase pi
GSTT: Glutathione S-transferase theta
OR: Odds ratio
PCR: Polymerase chain reaction
PPARGC1A: Peroxisome proliferator-activated receptor gamma coactivator 1-alpha
ROC: Receiver operating characteristic
ROS: Reactive oxygen species
SPSS: Statistical package for the social sciences
TGS: Total genotype score
UCI: Union cycliste internationale
